# Genomic Characterization of Uropathogenic *Escherichia coli* Isolates from Tertiary Hospitals in Riyadh, Saudi Arabia

**DOI:** 10.3390/ijms24087582

**Published:** 2023-04-20

**Authors:** Rawan H. Aljohani, Dalia S. ElFeky, Abdulrahman A. Alswaji, Eisa Alrashidi, Liliane Okdah, Bassam Alalwan, Sameera M. Aljohani, Hanan H. Balkhy, Alya Redhwan, Majed F. Alghoribi

**Affiliations:** 1 Infectious Diseases Research Department, King Abdullah International Medical Research Center, Riyadh 11481, Saudi Arabia; 2 Department of Biology, College of Science, Princess Nourah bint Abdulrahman University, Riyadh 11564, Saudi Arabia; 3 Department of Basic Medical Sciences, College of Medicine, Princess Nourah bint Abdulrahman University, Riyadh 11564, Saudi Arabia; 4 Department of Medical Microbiology and Immunology, Faculty of Medicine, Cairo University, Cairo 12613, Egypt; 5 Department of Pathology and Laboratory Medicine, King Abdulaziz Medical City (KAMC), Ministry of National Guard Health Affairs (MNGHA), Riyadh 11426, Saudi Arabia; 6 Department of Basic Science, College of Science and Health Professions, King Saud bin Abdulaziz University for Health Sciences, Riyadh 14611, Saudi Arabia; 7 World Health Organization, Geneva 1211, Switzerland; 8 Department of Health, College of Health and Rehabilitation Sciences, Princess Nourah bint Abdulrahman University, Riyadh 11564, Saudi Arabia

**Keywords:** uropathogenic *E. coli*, urinary tract infection, antimicrobial resistance, molecular typing, whole-genome sequence, extended-spectrum β-lactamases, Saudi Arabia

## Abstract

Uropathogenic *Escherichia coli* (UPEC) is the most common cause of urinary tract infections (UTIs) in hospitalised and non-hospitalised patients. Genomic analysis was used to gain further insight into the molecular characteristics of UPEC isolates from Saudi Arabia. A total of 165 isolates were collected from patients with UTIs between May 2019 and September 2020 from two tertiary hospitals in Riyadh, Saudi Arabia. Identification and antimicrobial susceptibility testing (AST) were performed using the VITEK system. Extended-spectrum β-lactamase (ESBL)-producing isolates (n = 48) were selected for whole genome sequencing (WGS) analysis. In silico analysis revealed that the most common sequence types detected were ST131 (39.6%), ST1193 (12.5%), ST73 (10.4%), and ST10 (8.3%). Our finding showed that *bla*_CTX-M-15_ gene was detected in the majority of ESBL isolates (79.2%), followed by *bla*_CTX-M-27_ (12.5%) and *bla*_CTX-M-8_ (2.1%). ST131 carried *bla*_CTX-M-15_ or *bla*_CTX-M-27_, and all ST73 and ST1193 carried *bla*_CTX-M-15_. The relatively high proportion of ST1193 in this study was notable as a newly emerged lineage in the region, which warrants further monitoring.

## 1. Introduction

Urinary tract infections (UTIs) are the most common infection worldwide, with considerable economic consequences, morbidity, and mortality. UTIs cause inflammation of the urethra (urethritis), bladder (cystitis), and kidney (pyelonephritis) [1]. The infection affects individuals regardless of age and gender; however, its incidence is highest in females owing to the physiological and structural characteristics of the urethra [2]. 

Uropathogenic *Escherichia coli* (UPEC) is the most common cause of UTIs in hospitalised and non-hospitalised patients [3]. UPEC strains harbour numerous virulence factors that contribute to their ability to cause disease. UPEC strains can be distinguished from other *E. coli* pathotypes by specific virulence-associated determinants, including diverse adhesins, toxins, siderophores, capsule variants, and other miscellaneous traits [4]. Molecular typing methods are used to differentiate and characterise UPEC strains from each other by advanced molecular investigation methods. Such methods include multilocus sequence typing (MLST) and WGS [5,6]. These methods have been used to type UPEC strains isolated from clinical specimens and can be used in combination to elucidate the global epidemiology of UPEC strains [7].

Given their importance, it is imperative to understand the major lineages of UPEC and their role in the global dissemination of high-risk pathogens. The identification and characterisation of different UPEC lineages are essential for improving the understanding, diagnosis, and treatment of UTIs. One of the major lineages of UPEC that is of particular concern is ST131, which is responsible for a large percentage of UTIs and bloodstream infections [8]. This clone has been studied extensively to gain insight into its pathogenesis, antimicrobial resistance (AMR), and epidemiology [9]. ST131 is typically associated with high AMR and is considered one of the most successful and vital lineages. It is characterised by its ability to acquire and spread plasmids and can acquire resistance to multiple antimicrobials, including extended-spectrum cephalosporins and carbapenems. Recently, ST1193 has been identified as an emerging lineage of UPEC responsible for causing UTIs and bloodstream infections [10]. This clone is following in the footsteps of ST131, which has been reported previously as the second most frequent clone among ESBL and fluoroquinolone-resistant *E. coli* isolates [11,12,13,14]

In Saudi Arabia, several studies have reported an increased frequency of AMR and the prevalence of UPEC, which requires urgent attention to understand the phenotypic and genotypic traits [15,16,17]. Studies examining the molecular mechanisms of UPEC are lacking in Saudi Arabia. Here, we utilised WGS analysis to perform genomic characterization of UPEC isolates recovered from two hospitals in Riyadh, Saudi Arabia, namely, King Abdul-Aziz Medical City (KAMC) and King Abdullah bin Abdul-Aziz University Hospital (KAAUH).

## 2. Results

### 2.1. Isolate Demographics 

The majority of UTIs in this study were community-acquired (n = 107/165, 64.8%), followed by hospital-acquired UTIs (n = 58/165, 35.2%), and these frequencies were consistent between the two hospitals. Female patients were predominant in both; KAAUH (76.9%) and KAMC (86%). The mean age of patients was 39.8 ± 26.4 (mean ± SD), composed of 20% of seniors (≥65 years), 50.3% of adults (25 to 64 years), 9.1% of youth (from 15 to 24 years) and 20.6% children (≤14 years).

### 2.2. Antimicrobial Susceptibility Testing

The antimicrobial susceptibility of the examined strains to different antimicrobial agents was routinely tested in the clinical microbiology laboratories at KAAUH and KAMC. Among the 165 isolates, 56 (33.9%) were ESBL-producing *E. coli* isolates, of which 22% (n = 22/100) were collected from KAAUH and 52.3% (n = 34/65) were collected from KAMC. The percentage of resistance to the tested antibiotics is shown in Table 1. Ciprofloxacin resistance was detected in 35.8% (n = 59/165) of isolates, 35 of which were ESBL-producing. All isolates examined in this study were susceptible to tigecycline and carbapenem (imipenem and meropenem).

### 2.3. Phylogenetic Group and MLST

In this study, WGS was conducted on 48 of 165 isolates, which represent 18% (18/100) of ESBL-producing UPEC isolates from KAAUH, and 46.2% (30/65) KAMC. Phylogenetic grouping analysis revealed that 48 ESBL isolates belonged to 1 of 7 groups, 62.5% were B2 (n = 30/48), 16.6% were A (n = 8/48), 8.3% were B1 (n = 4/48), 6.2% were D (n = 3/48), and groups C, F, and G (newly reported phylogroup) were represented by a single isolate (n = 1/48, 2.1%). The in silico MLST analysis revealed 16 different STs, including major lineages of UPEC. At 39.6% (n = 19/48), ST131 clonal complex was the most detected among the sequence isolates in this study, followed by ST1193 with 12.5% (n = 6/48), ST73 with 10.4% (n = 5/48), ST10 with 8.3% (n = 4/48), and ST405 and ST69 with 4.2% each (each n = 2/48) (Table 2). Among the ST131, three KAAUH isolates with a single-locus variant (SLV) fumC (~40) allele difference and one KAMC isolate with SLV PurA (355) allele difference were detected. Other singleton STs were detected in both hospitals, as shown in Table 2. All ST131, ST1193, and ST73 strains belonged to phylogenetic group B2 and ST10 with 8.3% (n = 4/48) and 4.2% of ST450 (n = 2/48) belonged to group A. ST4496, ST4380, ST443, and ST5614 belonged to group B1. All 4.2% ST69 (n = 2/48) and 2.1% ST38 (n = 1/48) belonged to group D. As for single isolates, ST1163 belonged to group G, ST88 belonged to group C, and ST62 belonged to group F.

### 2.4. Serotyping and FimH Typing 

Serotyping and *FimH* typing were performed for the ESBL-sequenced isolates. The most prevalent ST131 isolates made up 39.6% (n = 19/48) of the sample, with 57.9% (n = 11/19) were associated with O25:H4-*fimH30*, 26.3% (n = 5/19) with O16:H5 *fimH41* and 15.8% (n = 3/19) with O16:H5-*fimH141*. The second most prevalent ST1193 isolates made up 12.5% (n = 6/48) of the sample and were associated with included O75:H5- *fimH64* (n = 4/6), and two isolates had no O serotype. ST73 isolates were mostly associated with O6:H1- *fimH30* (n = 3/5). Serotypes and *fimH* typing for the other isolates varied across STs.

### 2.5. Detection of ESBL Genes

In silico detection of resistance genes was performed based on WGS sequencing data, adopting Abricate and PointFinder tools. Ten antibiotic classes were detected: aminoglycosides, β-lactams, chloramphenicol, lincosamide, macrolides, quinolone, streptomycin, sulfonamide, tetracycline, and trimethoprim. 

All the ESBL-producing UPEC strains carried 1 or more resistance genes, 38 (79.2%) of which harbour 3 or more resistance genes to different antibiotic classes. The majority of the ESBL-producing UPEC isolates in this study carried class A ESBL, including *bla*_CTX-M-15_ (n = 38/48, 79.2%), followed by *bla*_CTX-M-27_ (n = 6/48, 12.5%) and *bla*_CTX-M-8_ (1/48, 2.1%). The *bla*_CTX-M-15_ gene was detected in the majority of the STs but was missing in all the O16:H5-ST131 *fimH*141 isolates in this study; these isolates carried *bla*_CTX-M-27_. The *bla*_CTX-M-8_ was only detected in one isolate, which belonging to ST10 (Table 3). In addition, class C β-lactamases genes were detected, including *bla*_EC-5_ (n = 31/48, 64.6%), *bla*_EC-8_ (n = 3/48, 6.3%), *bla*_EC_ (n = 4/48, 8.3%), *bla*_EC-15_ (n = 4/48, 8.3%), *bla*_EC-18_ (n = 4/48, 8.3%), and *bla*_EC-13_ (n = 1/48, 2.1%). All isolates belonging to major UPEC lineages ST73, ST131, and ST1193 carried *bla*_EC-5,_ whereas the other *bla*_EC-like_ were detected in less frequent STs (Table 3).

### 2.6. Detection of QRDR Mutations and PMQR Genes

About 77.1% (n = 37/48) of the ESBL isolates in this study were resistant to fluoroquinolones. Quinolone resistance-determining regions (QRDRs) were investigated to detect chromosomal mutations in *gyrA*, *parC*, and *parE*, as shown in Table 4. The most prevalent mutations included *gyrA* (p.S83L) (n = 38/48, 79.2%), *gyrA* (p.D87N) (n = 30/48, 62.5%), and *parC* (p.S80I) (n = 33/48, 68.8%), which were detected in most of the major STs, except ST73. Other less frequent mutations were mainly detected in ST131, including *parC* (p.E84V) and *parE* (p.I529L), whereas all the O16:H5-ST131 *fimH141* (n = 3) isolates have the same mutations pattern (Table 4). Similarly, all ST1193 *fimH64* (n = 6) isolates have shown a pattern of mutations in *gyrA*, *parC* and *parE*. Notably, the mutation rate of *parE* among the major STs was higher than other STs, particularly *parE* (p.I529L), which was significantly associated with ST131, and *parE* (p.L416F), which was significantly associated with ST1193. In addition, seven isolates harboured plasmid-mediated quinolone resistance (PMQR) genes (five *qnrS1* and one *qnrB7*), which were detected in less frequent STs (Table 4).

### 2.7. Prevalence of Virulence Factors

Carriage of virulence factors in the ESBL-producing isolates was assessed using sequence data. The most frequently identified UPEC virulence factors (100%) in all the sequenced isolates were factor adherence *E. coli* (*fdeC*), curli fibres (csgG), siderophore enterobactin (*entABCDEF*, *fepABCDG* and *fes*), and outer membrane protein A (OmpA). Almost all isolates (n = 47, 97.9%) belonged to phylogroup B1, B2, G and F harbouring virulence genes encoding *E. coli* common pilus (*ecpABDER*) except *ecpC* (n = 44, 93.8%). Type I fimbriae (*fimACDEFGHI*) were found in 95.8% (n = 46/48) isolates, with only two isolates belonging to phylogroup A (n = 1 ST450, and n = 1 ST617) collected from KAAUH. In addition, the *fimB* gene was detected in 72.9% (n = 35/48) isolates and was missing in isolates belonging to phylogroup B2 (n = 10 ST131, n = 1 ST73) and phylogroup A (n = 1 ST450, n = 1 ST617). 

ST131 was significantly associated with several virulence factors, including *E. coli* heme uptake (*chuASTUVWXY*, *p* < 0.0005), afimbrial adhesin AFA-I (*afaABCD*, *p* < 0.02), Afa/Dr family (*daaF*, *p* < 0.007), Dr adhesins (*drap*, *p* < 0.007), yersiniabactin siderophore (*fyuA*, *irp12*, *ybtAEPQST*, *p* < 0.007), aerobactin (*iucAB*, *p* < 0.02) and *p* fimbriae (*papB p* = 0.04 and *papIX p* = 0.0001). ST1193 was significantly associated with the K1 capsule (*kpsT*, *p* = 0.0001), vacuolating autotransporter (*vat*, *p* = 0.0008), enterotoxin (*senB p* = 0.004) and *p* fimbriae (*papB*, *p* = 0.02). ST73 was significantly associated with the salmochelin siderophore (*iroBCDEN*, *p* = 0.0004), F1C fimbriae (*focAG*, *p* < 0.009 and *focCD*, *p* = 0.0006), exotoxin hemolysin (*hlyABD p* = 0.005), S fimbriae (*sfaBC p* = 0.001 and *sfaDGHY p* = 0.009), effector delivery system (*pic*, *p* = 0.001) and immune modulation (*tcpC*, *p* = 0.001). ST10 expression was significantly associated with TTSS secreted effectors (*espL1 p* = 0.01, *espX4 p* = 0.003, *espL4 p* = 0.0006 and *espY1 p* = 0.008). 

### 2.8. SNP-Based Phylogenetic Analyses

**Figure 1:**
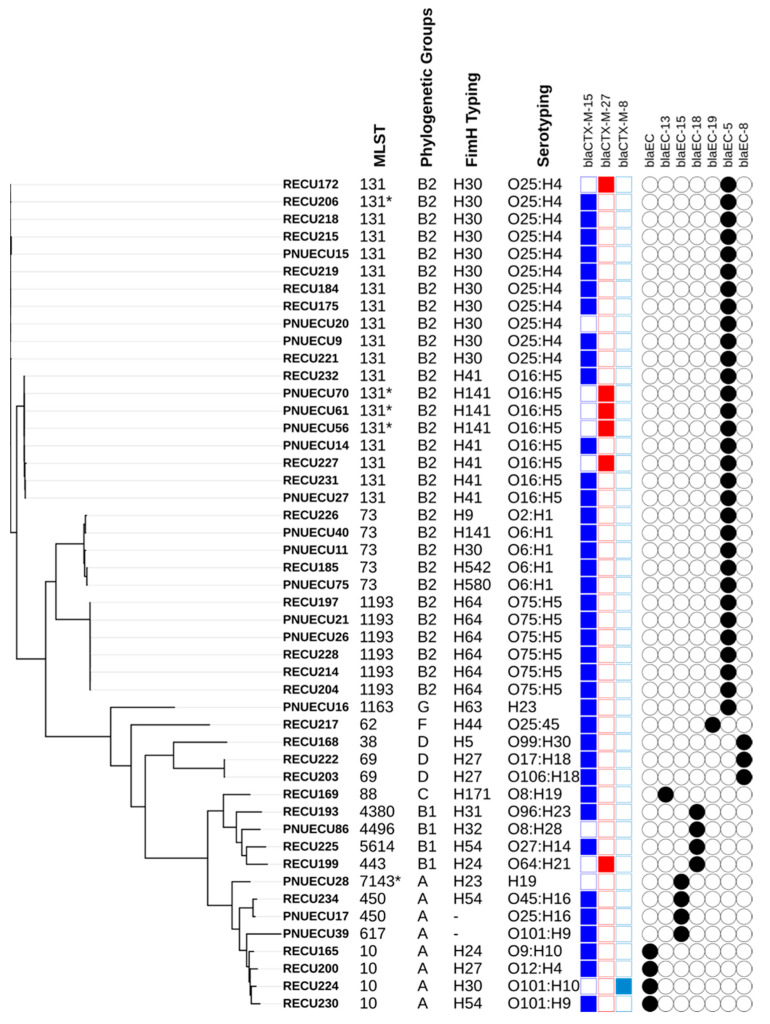
SNP-derived phylogenetic tree of the UPEC ESBL strains in this study, compared with MLST, phylogenetic groups, fimH typing, serotyping, and ESBL resistance genes. * Isolates displaying a single-locus variant (SLV).

The ESBL isolates in this study were separated into several groups, which were aligned using MLST, phylogenetic groups, *fimH* typing, and serotyping. ST131, ST73, and ST1193 were found in a clade representing phylogenetic group B2. ST131 was a major lineage of UPEC (n = 19) and was separated primarily into three subclades: O25b-H4-ST131-fimH30/clade, O16-H5-ST131-*fimH*41/clade, and O16-H5-ST131-*fimH141.* As shown in Figure 1, ST131 harboured *bla*_CTX-M-15_ and *bla*_CTX-M-27_. Notably, *bla*_CTX-M-27_ was primarily detected in ST131; however, it was also seen in ST443. All O16-H5-ST131 *fimH141* isolates carried only *bla*_CTX-M-27_, which warrants further investigation. ST73 was found in one clade, and all clades harboured *bla*_CTX-M-15_; however, ST73 showed different *fimH* typing and two serotypes. As a newly emerged ESBL lineage, ST1193 found in one clade harboured *bla*_CTX-M-15_ with the same phylogenetic grouping (B2), *fimH64*, and serotyping O75:H5. The other STs were separated into different branches on the phylogenetic tree associated with different traits (Figure 1).

## 3. Discussion

In this study, we investigated the prevalence, clonal relatedness, and antibiotic susceptibility of UPEC isolates from patients with UTIs at two tertiary care hospitals in Riyadh, Saudi Arabia. We used both phenotypic and genotypic methods to identify ESBL-producing *E. coli* isolates and found that 33.9% (56/165) of the UPEC isolates tested positive for ESBL. The ESBL-producing UPEC isolates were highly resistant to other commonly used antibiotics, such as ciprofloxacin and trimethoprim.

Our findings showed the prevalence of ESBL-producing UPEC (33.9%) in both KAAUH and KAMC, which was nearly consistent with other reports from Saudi Arabia that found 35% in 2015 [15] and 33% in 2018 [16]. However, the prevalence of ESBLs in KAMC increased from 35% in the isolates collected for investigations in 2012–2013 to 51.5% in the isolates gathered for this study in 2019–2020. [15]. This poses major healthcare and economic burdens that lead to serious and complicated UTIs with limited treatment options. Our study aimed to enhance the AMR WGS-based surveillance network to monitor the spread of UPEC within Riyadh city and among different hospitals. 

This study characterised 48 ESBL-producing UPEC isolates using the WGS approach. In silico investigation was performed using the generated sequences in this study to determine the clonal structure of the examined isolates by means of phylogrouping and serotyping, followed by MLST and SNP-derived phylogenetic analysis, to determine the clonal relatedness. Our findings showed that the majority of the ESBL-producing UPEC isolates in our study (n = 30/48, 62.5%) belonged to phylogenetic group B2. This is consistent with earlier UPEC findings in Saudi Arabia, which demonstrated the dominance of this group over other phylogenetic groups observed in this study, including A, B1, D, F, and G [15,17].

The results of this study revealed the presence of multidrug-resistant UPEC strains with high virulence potential, including ST10, ST69, ST73, ST131, ST405, and ST1193. ST131 was the most predominant sequence type in our study, which is a global pandemic clone responsible for community and hospital-acquired UTI and bloodstream infections [18,19,20]. This successful clone was first identified in 2008 as capable of producing ESBLs and is currently considered the most common multidrug-resistant, ESBL-producing UPEC strain worldwide [21,22,23,24].

The geographical distribution of ST131 has yet to be completely understood; however, it has been found in humans, animals, and food sources in Europe, North America, Canada, Japan, Korea, Asia, the Middle East, and Africa [7,23,25]. Subsequent research confirmed the worldwide prevalence of ST131 and its wide range of virulence and resistance genes present in transferable plasmids [26,27]. Additionally, ST131 is highly prevalent amongst fluoroquinolone-resistant *E. coli* [9].

It is clear that ST131 is highly diverse and can vary significantly in terms of AMR and virulence. This clone has been identified as the primary cause of UTIs in adults and children worldwide, partly due to its ability to develop resistance to a broad range of antibiotics readily. Furthermore, ST131 is known to be involved in outbreaks of healthcare-associated UTIs that have high antibiotic resistance, making them challenging to treat [28,29,30].

The molecular phylogeny of *E. coli* ST131 was studied, and three major subclades were revealed: A, B, and C. The ST131 subclone delineation is primarily based on *fimH* alleles, serotypes, and the carriage of AMR genes [7,31]. Our finding divided ST131 into two clades, clade A (*fimH41* and *fimH141*) and clade C (*fimH30*). ST131 *fimH30* isolates are typically serotype O25:H4, while *fimH41* and *fimH141* belong to serotype O16:H5. 

The carriage of AMR genes also contributes to the distinction between the clades. The O25:H4-ST131 *fimH30* subclone is a major global concern and serves as a background for simultaneous resistance to multiple drugs with different mechanisms of action and resistance. The O25:H4-ST131 *fimH30* clade C2 (also known as C2-*H30*Rx) is rapidly expanding, associated with the production of CTX-M-15 and fluoroquinolone resistance [21,32,33]. This subclone was the most detected ST131 in our study, associated with a high resistance rate, as illustrated in Table 3 and Table 4. Moreover, we have observed that other subclones of ST131, including C1-M27, are associated with the production of CTX-M-27. The C1-M27 was first detected during the late 2000s among ESBL-producing *E. coli* in Japan [24,34,35]. Along with C1-M27, *bla*_CTX-M-27_ gene was detected in other ST131 subclones, including *fimH41* and *fimH141*. Each of these subgroups has its own unique genetic markers and phenotypic traits.

Studies have found that carbapenem-resistant strains of *E. coli* ST131 have emerged in recent years because of the acquisition of plasmids carrying carbapenemase encoding genes, such as *bla*_OXA-48,_ *bla*_NDM,_ and *bla*_KPC–2_ [30,36,37]. Therefore, it is crucial to be aware of the potential risks associated with this clone and take measures to prevent its spread. 

Currently, ST1193 is an emerging multidrug-resistant clone rapidly spreading worldwide, mimicking the success of the highly successful ST131 clone [10]. It was first reported in 2012 among fluoroquinolone-resistant *E. coli* isolates recovered from humans and dogs in Australia between 2007 and 2008 [10]. ST1193 evolved from clonal complex (CC) 14 in the early 1990s through the transition of type 1 pili from *fimH27* to *fimH64* and the acquisition of three QRDR mutations in *gyrA* (p.S83L and p.D87N), *parC* (p.S80I), and *parE* (p.L416F) [10,13]. To the best of our knowledge, this is the first report of ST1193 *fimH64* in Saudi Arabia carrying QRDR mutations and β-lactamase genes (*bla*_CTX-M-15_ and *bla*_EC-5_). This clone has been reported in various countries and is associated with serious infections, including urinary tract and bloodstream. To prevent the further spread of this clone, it is important to use effective antibiotic stewardship and infection control measures and improve the surveillance and identification of high-risk clones.

In conclusion, we described the genomic characterisation of UPEC isolates recovered from two hospitals in Riyadh, Saudi Arabia. WGS has been proven to be a powerful epidemiological tool for investigating UPEC [6]. Members of the ST131 lineage constitute a key UPEC clone, which, in our study, was unusually associated with high virulence in addition to broad antibiotic resistance. ST1193 is a recently evolving lineage that can carry *bla*_CTX-M-15_ and warrants close monitoring. Further studies are required to limit the spread of major UPEC lineages that can display high virulence potential and a broad spectrum of drug resistance, including the recent dissemination of carbapenemase genes such as *bla*_NDM_ and *bla*_OXA_ [38,39,40].

## 4. Materials and Methods 

### 4.1. Bacterial Isolates and Phenotypic Testing

Non-duplicate E. coli UPEC isolates (n = 165) were recovered from patients with UTIs from two tertiary care hospitals in Riyadh, Saudi Arabia, KAMC (n = 65) and KAAUH (n = 100), from May 2019 to September 2020. Demographic data of the study participants were obtained from each hospital’s electronic medical record system. UPEC isolates were collected from the clinical microbiology laboratories of each hospital after routine diagnostics. Patient demographics, including gender, age, and hospitalisation details, were retrieved from the medical record system. Bacterial identification and antimicrobial susceptibility testing were performed using the VITEK II instrument (BioMerieux, Marcy-l’Etoile, France).

### 4.2. Whole Genome Sequencing 

Approximately one-third of the UPEC isolates (n = 48/165, 29.1%) were selected for whole genome sequencing to represent most ESBL-producing isolates collected in this study. Genome sequencing was performed using the MiSeq Illumina platform with a 2 × 300 bp paired-end reads protocol. Prior to sequencing, DNA was extracted using the QIAamp DNA Mini Kit (QIAGEN, Hilden, Germany) and a Qubit Fluorometric Quantitation Thermo Fisher (Invitrogen, Waltham, MA, USA) was used to measure DNA quantity and integrity. According to the manufacturer’s instructions, DNA library preparation was performed using the Nextera XT DNA Library Prep Kit (Illumina, Cambridge, UK). 

### 4.3. Bioinformatic Analysis

The generated sequence reads were first assessed using the FastQC tool (v.0.11.8), assembled using Unicycler (version 0.4.8) [5] and annotated using Prokka (version 1.14.6) [6] with default parameters. The presence of resistance genes, virulence factors and multilocus sequence typing (MLST), serotype, and *FimH* type were determined using Abricate (version 0.9.8) “https://github.com/tseemann/abricate (accessed on 19 January 2023)” with the appropriate databases, i.e., NCBI AMRFinderPlus [41], virulence factor database (VFDB) [42] and mlst “https://github.com/tseemann/mlst (accessed on 19 January 2023)”, SerotypeFinder (version 1.0) [43], and FimTyper (version 1.0) [44] from the Center for Genomic Epidemiology (CGE) “http://genomicepidemiology.org/services (accessed on 19 January 2023)”. The phylogenetic typing (phylogroups) was performed on all WGS data of ESBL-producing *E. coli* using the Clermont typing tool “http://clermontyping.iame-research.center/ (accessed on 19 January 2023)”, which identifies a Clermont phylogenetic type (A, B1, B2, C, D, E, F and G) for each sequence. Chromosomal mutations defining quinolone resistance were analysed using PointFinder [45]. SNP-based phylogenetic analysis with default options was performed using Snippy (version 3.1.0) “https://github.com/tseemann/snippy (accessed on 19 January 2023)”.

## Figures and Tables

**Table 1:** Percentage of resistance to antibiotics among the UPEC isolates studied.

Antimicrobial Category	Antimicrobial Agents	KAAUH	KAMC	All
		n = 100 (%)	n = 65 (%)	n = 165 (%)
β-lactams	Ampicillin	56 (56)	53 (81.5)	109 (66.1)
	Amoxicillin-clavulanate	9 (9)	4 (6.2)	13 (7.9)
	Piperacillin/Tazobactam	5 (5)	2 (3.1)	7 (4.2)
	Cefalotin	34 (34)	34 (52.3)	68 (41.2)
	Cefoxitin	2 (2)	5 (7.7)	7 (4.2)
	Ceftazidime	9 (9)	19 (29.2)	28 (17)
	Cefepime	4 (4)	9 (13.8)	13 (7.9)
	Ceftriaxone	21 (21)	33 (50.8)	54 (32.7)
Fluoroquinolones	Ciprofloxacin	21 (21)	38 (58.5)	59 (35.8)
Aminoglycosides	Amikacin	0 (0)	1 (1.5)	1 (0.6)
	Gentamicin	7 (7)	10 (15.4)	17 (10.3)
Sulfamethoxazole	Trimethoprim/Sulfamethoxazole	34 (34)	41 (63.1)	75 (45.5)
Others	Nitrofurantoin	3 (3)	3 (4.6)	6 (3.6)

**Table 2:** Prevalence of ESBL STs in KAAUH and KAMC.

ST	Phylogroup	KAAUH	KAMC	All
		n = 18 (%)	n = 30 (%)	n = 48 (%)
ST131 *	B2	8 (44.4)	11 (36.7)	19 (39.6)
ST1193	B2	2 (11.1)	4 (13.3)	6 (12.5)
ST73	B2	3 (16.7)	2 (6.7)	5 (10.4)
ST10	A	-	4 (13.3)	4 (8.3)
ST450	A	1 (5.6)	1 (3.3)	2 (4.2)
ST69	D	-	2 (6.7)	2 (4.2)
ST617	A	1 (5.6)	-	1(2.1)
ST1163	G	1 (5.6)	-	1(2.1)
ST443	B1	-	1 (3.3)	1(2.1)
ST4496	B1	1 (5.6)	-	1 (2.1)
ST38	D	-	1 (3.3)	1 (2.1)
ST62	F	-	1 (3.3)	1 (2.1)
ST88	C	-	1 (3.3)	1 (2.1)
ST4380	B1	-	1 (3.3)	1 (2.1)
ST5614	B1	-	1 (3.3)	1 (2.1)
ST7143 *	A	1 (5.6)	-	1 (2.1)

* Isolates displaying single-locus variant (SLV).

**Table 3:** Sequence types and prevalence of β-lactamases genes identified in UPEC isolates from this study.

ST (n)	β-Lactamase Genes			Class C β-Lactamases Genes						
	*bla*_CTX-M-15_ (%)	*bla*_CTX-M-27_ (%)	*bla*_CTX-M-8_ (%)	*bla*_EC_ (%)	*bla*_EC-5_ (%)	*bla*_EC-8_ (%)	*bla*_EC-13_ (%)	*bla*_EC-15_ (%)	*bla*_EC-18_ (%)	*bla*_EC-19_ (%)
O25:H4-ST131 *fimH30* (11)	9 (81.8)	1 (9.1)	-	-	11 (100)	-	-	-	-	-
O16:H5-ST131 *fimH41* (5)	4 (80)	1 (20)	-	-	5 (100)	-	-	-	-	-
O16:H5-ST131 *fimH141* (3)	-	3 (100)	-	-	3 (100)	-	-	-	-	-
ST1193 *fimH64* (6)	6 (100)	-	-	-	6 (100)	-	-	-	-	-
ST73 (5)	5 (100)	-	-	-	5 (100)	-	-	-	-	-
ST10 (4)	3 (75)	-	1 (25)	4 (100)	-	-	-	-	-	-
ST69 (2)	2 (100)	-	-	-	-	2 (100)	-	-	-	-
ST405 (2)	2 (100)	-	-	-	-	-	-	2 (100)		
Other STs (10)	7 (70)	1 (10)	-	-	1 (10)	1 (10)	1 (10)	2 (20)	4 (4)	1 (10)
Total (48)	38 (79.2)	6 (12.5)	1 (2.1)	4 (8.3)	31 (64.6)	3 (6.3)	1 (2.1)	4 (8.3)	4 (8.3)	1 (2.1)

**Table 4:** Sequence types and prevalence of QRDR mutations and PMQR genes in UPEC isolates from this study.

ST (n)	Vitek	QRDR Mutations										PMQR Genes (%)	
		*gyrA* (%)			*parC* (%)			*parE* (%)				*qnrS1*	*qnrB7*
	CIP-R	p.S83L	p.D87N	p.D87Y	p.S57T	p.S80I	p.E84V	p.I355T	p.L416F	p.S458A	p.I529L		
O25:H4-ST131 *fimH30* (11)	10 (90.9)	11 (100)	9 (81.8)	-	-	11 (100)	9 (81.8)	-	-	-	9 (81.8)	-	-
O16:H5-ST131 *fimH41* (5)	4 (80)	4 (80)	1 (20)	1 (20)	-	2 (40)	1 (20)	-	-	-	4 (80)	-	-
O16:H5-ST131 *fimH141* (3)	3 (100)	3 (100)	3 (100)	-	-	3 (100)	3 (100)	-	-	-	3 (100)	-	-
ST1193 *fimH64* (6)	6 (100)	6 (100)	6 (100)	-	-	6 (100)	-	-	6 (100)	-	-	-	-
ST73 (5)	1 (20)	2 (40)	-	-	-	-	-	-	-	-	-	-	-
ST10 (4)	2 (50)	3 (75)	2 (50)	-	-	2 (50)	-	-	1 (25)	-	-	1 (25)	-
ST69 (2)	2 (100)	2 (100)	1 (50)	-	-	2 (100)	-	-	-	-	-	1 (25)	-
ST405 (2)	2 (100)	2 (100)	2 (100)	-	-	2 (100)	-	-	-	1 (50)	-	-	-
Other STs (10)	6 (60)	5 (50)	5 (50)	-	1 (10)	5 (50)	-	1 (10)	1 (10)	3 (30)	-	4 (40)	1 (10)
Total (48)	37 (77.1)	38 (79.2)	30 (62.5)	1 (2.1)	1 (2.1)	33 (68.8)	13 (27.1)	1 (2.1)	8 (16.7)	4 (8.3)	16 (33.3)	6 (12.5)	1 (2.1)

## Data Availability

The whole genome sequence of ESBL-producing UPEC isolates has been deposited in NCBI Sequence Read Archive (SRA) under accession numbers SRR22179269–SRR22179316. These sequences are part of BioProject no PRJNA897916.
